# A quality improvement project to assess the use of visual aids to improve understanding and motivation in periodontal patients

**DOI:** 10.1038/s41405-020-0041-9

**Published:** 2020-08-12

**Authors:** Pushpa Momin, Sophina Mahmood

**Affiliations:** 1 University of Manchester Dental Hospital, Manchester, UK; 2 Bradford Royal Infirmary, Bradford, UK

**Keywords:** Gum disease, Dental education, Gum disease

## Abstract

**Aims:**

A quality improvement project was carried out in a General Dental Practice in London. The aim was to improve understanding and motivation in periodontal patients.

**Methods:**

Research into interventions in the medical field to improve understanding and motivation amongst patients, lead to the idea of using visual aids to help motivate periodontal patients and to improve their understanding of the disease. The results from the first Plan-Do-Study-Act cycle using visual aids are presented.

**Results:**

After the use of visual aids, patients felt more motivated in maintaining good oral hygiene, felt the visual aids improved their understanding of gum disease and how to take care of their gums and teeth. One hundred percent of patients preferred their diagnosis to be explained to them with visual aids.

**Discussion:**

The project supported the increase in motivation and understanding in periodontal patients. However further data is required to learn more about how visual aids influence patient behaviour, how effective they are in improving patients’ understanding and motivation, and other factors which play a role in this.

**Conclusions:**

The results of this quality improvement project are promising; it has highlighted that visual aids can have a place in the management of periodontitis in general practice.

## Background

Severe periodontitis is the 6th most prevalent disease worldwide with a prevalence of 11.2% and around 743 million people affected globally.^1^

The first step in preventing periodontitis is by managing gingivitis through the maintenance of good oral hygiene. Patient understanding and motivation are essential in the management of any degree of periodontal disease. Motivated patients are aware of their own condition and more compliant with oral hygiene instructions (OHI), and therefore stabilisation of their disease. This is supported by a cross sectional study which concluded that periodontal patients with greater motivation, had better oral health, suggesting an influence on the quality of their self-management of the disease (i.e., complying to their oral hygiene regime).^2^

**Fig. 1:**
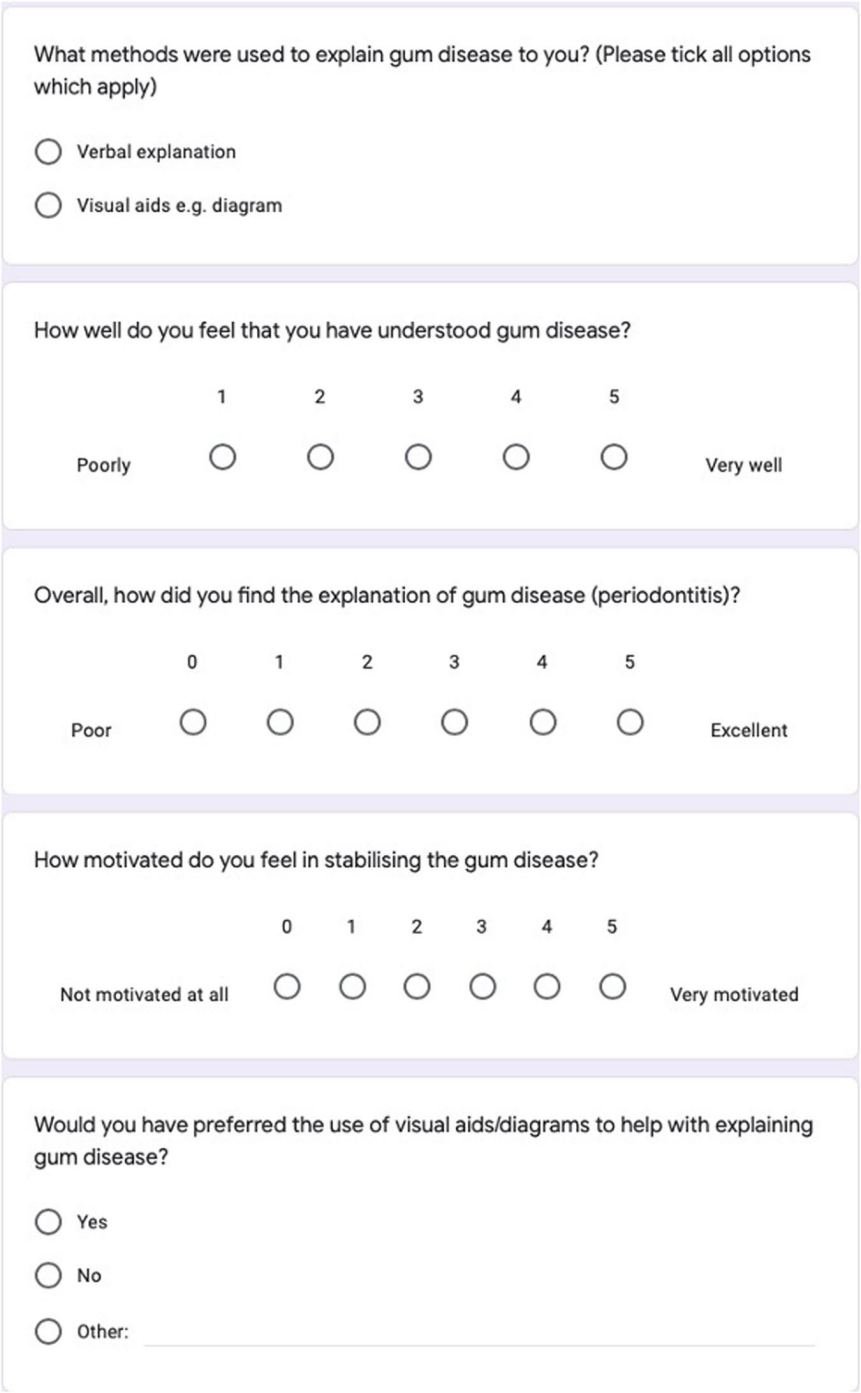
The survey given to patients before using visual aids. Five basic questions were asked to patients to provide information on their understanding and motivation.

**Fig. 2:**
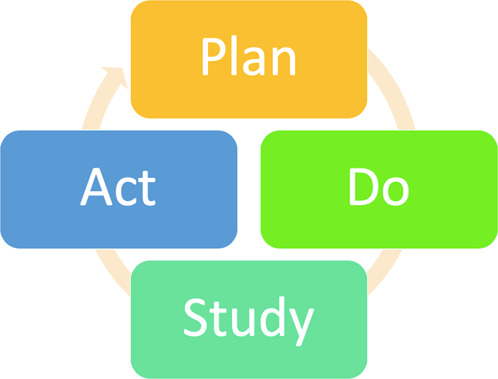
Plan-Do-Study-Act model. This concept allows a result from an intervention to be checked and modified, before final implementation.

**Fig. 3:**
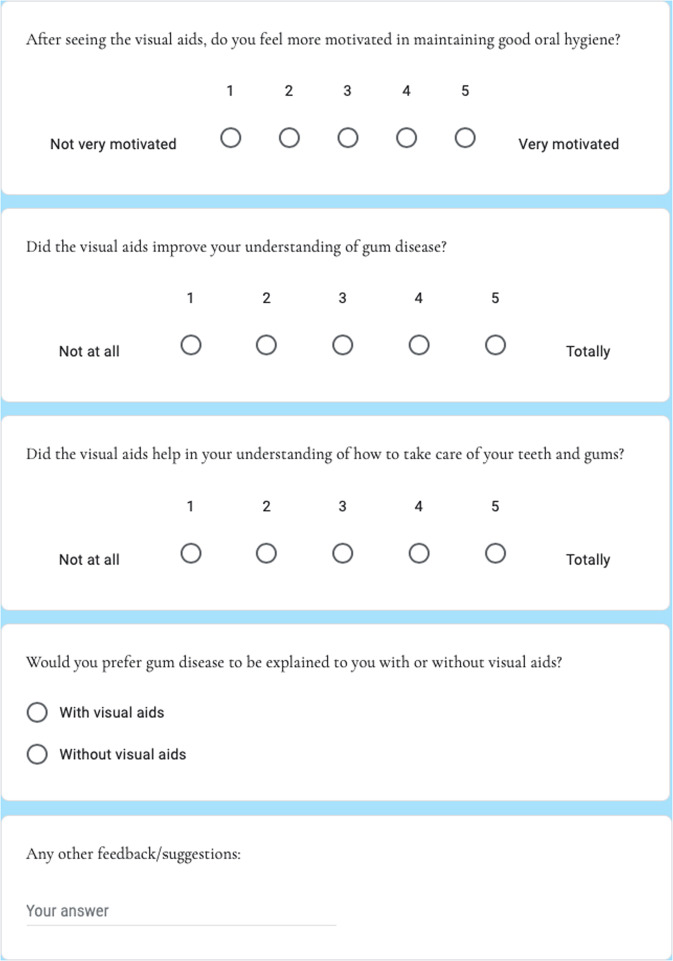
The survey given to patients after using visual aids. Four simple questions were asked as well as free text suggestions to provide further patient feedback.

**Fig. 4:**
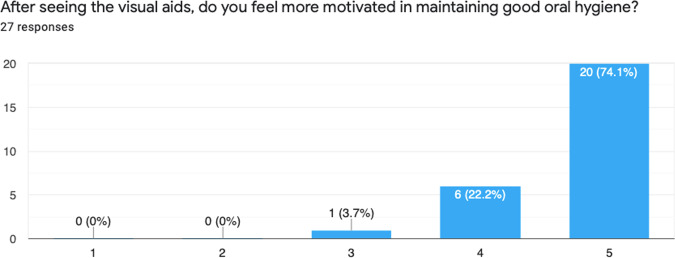
Results from Question 1. This graph demonstrates improved motivation levels for maintaining oral hygiene, after the use of visual aids.

**Fig. 5:**
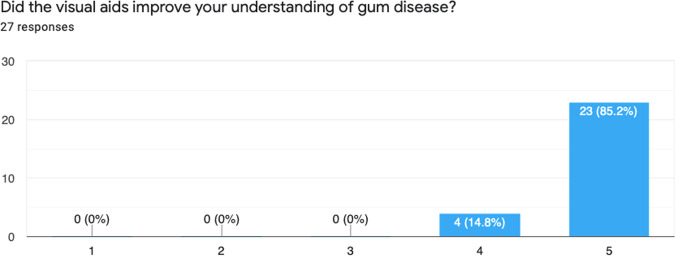
Results from Question 2. This graph illustrates improved understanding of periodontitis, after the use of visual aids.

**Fig. 6:**
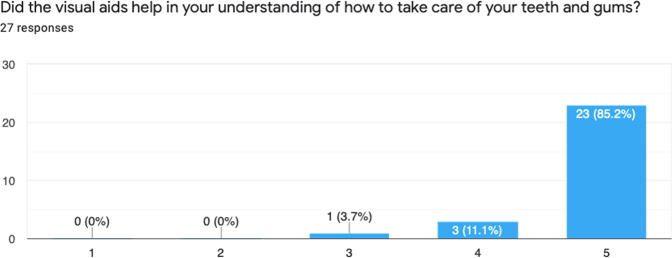
Results from Question 3. This graph demonstrates the improved understanding of how to maintain good oral hygiene, after the use of visual aids.

**Fig. 7:**
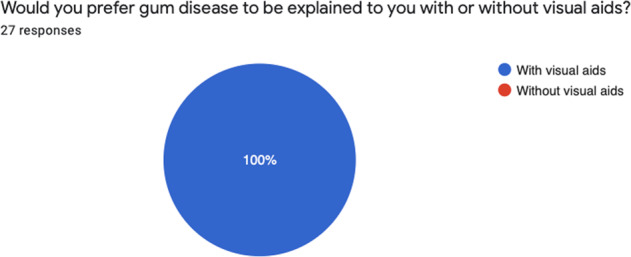
Results from Question 4. This chart shows patient's clear preference for the use of visual aids.

**Fig. 8:**
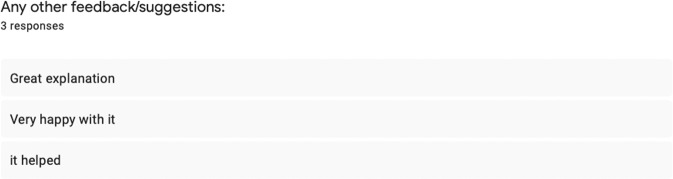
Feedback from patients. Free text comments were positive but limited in number.

The initiation of this quality improvement project was from an anecdotal observation; whilst working in general dental practice, the author noticed that there were several patients who have had multiple cycles of periodontal therapy with different clinicians. Through discussion, the patients showed very little understanding of their diagnosis and OHI, and lack of motivation in maintaining good oral hygiene.

Research into ways to improve patient understanding and motivation revealed several studies in medicine that support the use of visual aids in healthcare. Three randomised controlled trials demonstrated that using visual aids increases patient understanding in a medical setting.^3^ Furthermore, a systematic review concluded that visual aids tend to give rise to more enduring changes in attitudes and behavioural intentions, which can directly affect decision making and healthy behaviour.^4^

As these studies were centred around visual aids used within a medical context, the aim of this small quality improvement study was to investigate whether such methods could be applicable in dentistry.

The study was carried out in a general dental practice in London providing NHS and private care for patients in the local area.

Through visual aids, the study aimed to improve:

1. Patient motivation in maintaining good oral hygiene.
2. Patient understanding of periodontal disease and the importance of oral care.

In addition, the study also aimed to gain insight into any apparent barriers to the use of visual aids in the dental practice setting.

## Methods

A small pilot study in three dental practices in London was carried out; this involved a survey asking dentists whether they explain periodontitis using verbal explanations alone, or with adjuncts such as visual aids. In one of these practices, a further survey was given to 20 patients who had a diagnosis of periodontitis within the last 12 months (Fig. 1).

The pilot study highlighted a need for an intervention to improve patient motivation and understanding of periodontal disease. The findings from the pilot study will be discussed in the “*Results*” section.

Given the medical evidence supporting the use of visual aids, this intervention was tested in periodontal patients; the Plan-Do-Study-Act (PDSA) model of improvement was used to guide this process (Fig. 2).

### Plan

Visual aids were created, which included the aetiology of periodontitis, brushing technique and use of interdental brushes. QR codes were added, directing patients to YouTube videos demonstrating the aetiology of periodontitis and the modified bass technique.

### Do

Visual aid leaflets were provided to all dentists in this practice; a discussion took place to explain the potential benefits behind visual aids, exploration of any potential barriers which could be foreseen, and a discussion of how the visual aids should be used—with particular emphasis that they should supplement, not substitute, a verbal explanation. The visual aids were used with patients who have had a previous diagnosis of periodontitis. The visual aids were used over a 1 month period and 30 patients took the visual aid home along with a feedback survey.

A total of 27 patients took part in the project. Survey forms were given to each patient and they were requested to fill this out and return it to the dental practice reception (Fig. 3).

### Ethical considerations

As a service improvement, formal ethical approval was not required. All data collection was anonymous.

## Results

The pilot study revealed that all dentists were using verbal explanations alone to explain periodontitis. In the practice where a survey was given to 20 patients, the results revealed that 50% of patients did not feel they understood gum disease very well and 93% of patients would have preferred the use of visual aids. There were varying levels of motivation in patients to stabilise their disease, with 31% of patient’s expressing a lack of motivation.

The initial survey highlighted there was major room for improving the understanding and motivation of patients with periodontitis.

After introducing the intervention, 30 patients were given the visual aid and feedback survey, however 3 patients did not return the survey. Below is a summary of the results from the survey (Figs. 4–8).

## Discussion

The pilot study initially involved a survey of dentists at different dental practices in London to account for the possibility of varying methods of explaining periodontitis amongst different practices. The results from the pilot survey given to patients supported the anecdotal observations made by the authors, and identified a need for improving the understanding and motivation of patients with periodontitis. The pilot survey given to patients could be improved by including questions to ascertain the level of understanding, motivation and compliance to OHI.

The results of this initial pilot study lead to further research into ways of increasing patient understand and motivation, and the further study into visual aids. This quality improvement project has highlighted that using visual aids in general practice has been received well by periodontal patients and can play a role in patient education. It has also revealed several barriers in the use of visual aids in general practice.

**Fig. 9:**
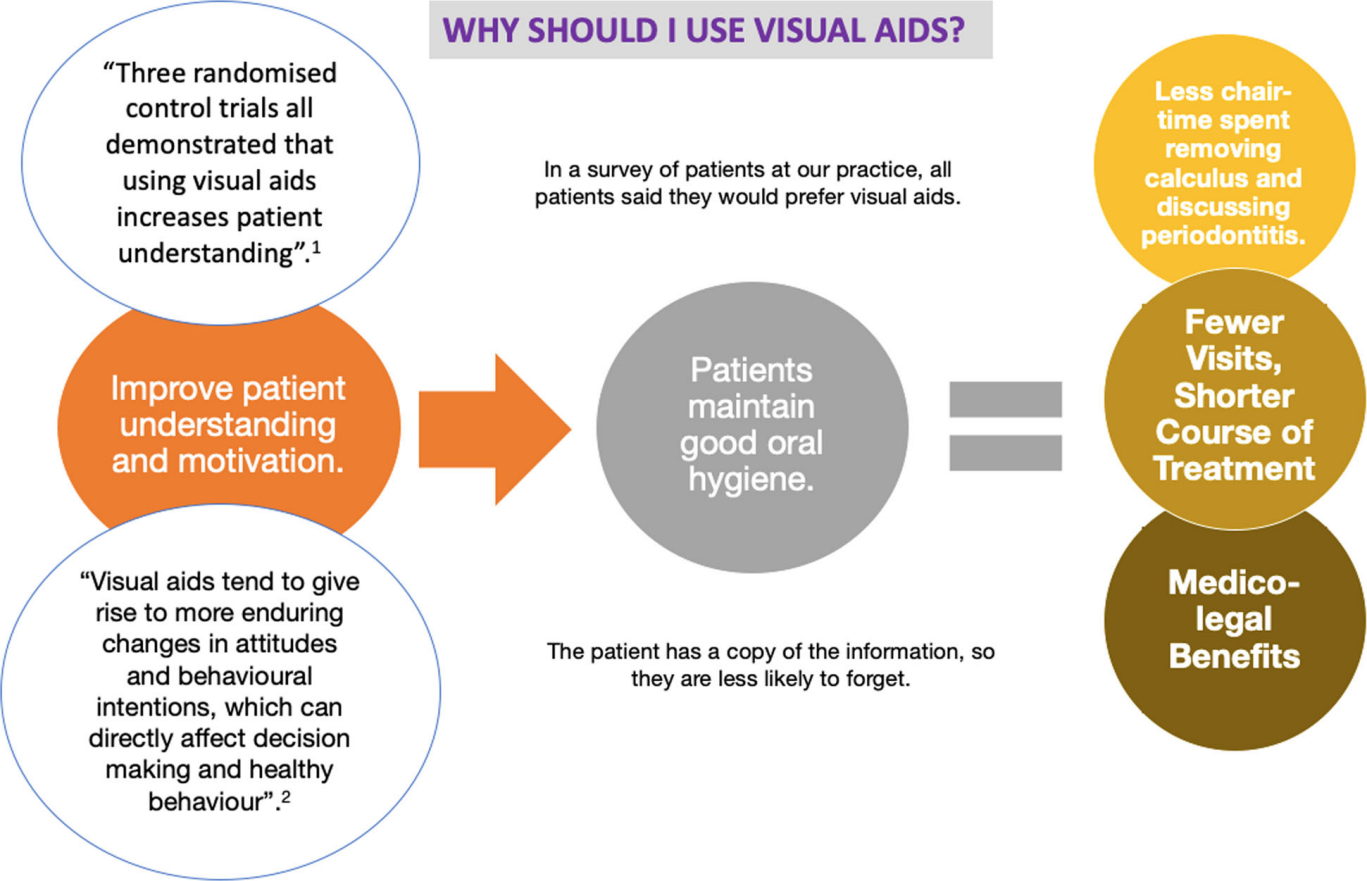
Poster provided to dentists. The key evidence and information was provided in a colourful format to engage and motivate dentists to use the visual aids. ^1^Ref. ^3^ and ^2^ref. ^4^.

When introducing the concept of visual aids to dentists; concern was raised that general dental practitioners are already under time pressure and using visual aids will increase chair-time with patients. Initially this may seem to be the case, however visual aids can potentially save time in the long run. If patients are more motivated and have a better understanding of OHI, less time can be spent in removing further deposits of calculus, and treatment is more likely to be successful. Further research would be needed to explore the average time taken to use visual aids and whether this improves oral hygiene over a period of time. Furthermore, if patients have a good understanding of their diagnosis from the onset, there is likely to be less repetition of this at future visits. A summary of these points was provided to dentists in a poster format (Fig. 9) and discussed during a practice meeting.

Many areas in the UK are increasingly diverse, particularly in London. This may introduce significant language and cultural barriers that hinder the effective delivery of preventive oral health care, especially where English is not the first language of the patient. Where possible, an interpreter should be arranged, particularly for appointments which involve explaining a diagnosis and consenting for treatment. Visual aids do not rely on fluency or comprehension in the English language, and therefore may aid the patient in understanding their diagnosis and implementing OHI. Furthermore, the videos which are linked to the QR codes on the visual aids, can be adjusted to link to oral health videos in different languages.

There are several limitations of this project. It would have been beneficial to have a larger patient base, and to explore the patients demographics and their experience of using the visual aids. Regular follow up of the patients would have been helpful to ascertain the effectiveness of the visual aids at intervals after the appointment that they were given.

The authors believe the wording used in the questionnaire could have been more patient-friendly. Piloting the questions on a set number of patients may have been beneficial to cater to varying levels of understanding in relation to oral health. In addition, the use of demonstrations of oral hygiene on models was overlooked as a visual aid. Providing a separate category for demonstrations on a model or in the patient’s mouth, would have provided a more accurate insight into how dentists are explaining OHI; this should be taken into consideration in future research.

There are several factors which influence patient understanding and motivation, such as socioeconomic status; ideally these should have been considered and may have provided further insight into the effectiveness in different patient groups. This project is just at the tip of the ice-berg. It has revealed the potential that visual aids have in periodontology, and highlighted areas of further research.

## Conclusion

The initial results of this quality improvement project have been promising, showing an increase in patient motivation in maintaining good oral hygiene, in understanding oral care, and a preference in all patients in using visual aids to help with explaining their diagnoses and preventative advice. Further data is required to learn more about how visual aids influence patient behaviour, how effective they are in improving patient’s understanding and motivation, and whether they have a place in the practice of periodontology. The use of visual aids are not likely to be limited to the management of periodontitis. If introduced prior to the development of disease, it can act as a preventative aid.

It is important to remember that maintaining good oral hygiene is only part of a multifaceted approach to periodontal treatment. Monitoring and management of risk factors play an important role and should not be overlooked. By developing tools and adjusting our practice, we can work towards improving outcomes for periodontal patients, and apply this to other areas within dentistry.
